# Shear-Coated Linear Birefringent and Chiral Cellulose Nanocrystal Films Prepared from Non-Sonicated Suspensions with Different Storage Time

**DOI:** 10.3390/nano11092239

**Published:** 2021-08-30

**Authors:** Olga Rubi Juárez-Rivera, Reina Araceli Mauricio-Sánchez, Kenneth Järrendahl, Hans Arwin, Arturo Mendoza-Galván

**Affiliations:** 1 Cinvestav-Unidad Querétaro, Libramiento Norponiente 2000, Querétaro 76230, Mexico; olga.juarez@cinvestav.mx (O.R.J.-R.); amauricio@cinvestav.mx (R.A.M.-S.); 2 Materials Optics, Department of Physics, Chemistry and Biology, Linköping University, SE-581 83 Linköping, Sweden; kenneth.jarrendahl@liu.se (K.J.); hans.arwin@liu.se (H.A.)

**Keywords:** cellulose nanocrystals, structural color, linear birefringence, circular dichroism, Mueller matrix

## Abstract

Nanocelluloses are very attractive materials for creating structured films with unique optical properties using different preparation techniques. Evaporation-induced self-assembly of cellulose nanocrystals (CNC) aqueous suspensions produces iridescent films with selective circular Bragg reflection. Blade coating of sonicated CNC suspensions leads to birefringent CNC films. In this work, fabrication of both birefringent and chiral films from non-sonicated CNC suspensions using a shear-coating method is studied. Polarization optical microscopy and steady-state viscosity profiles show that non-sonicated CNC suspensions (concentration of 6.5 wt%) evolve with storage time from a gel-like shear-thinning fluid to a mixture of isotropic and chiral nematic liquid crystalline phases. Shear-coated films prepared from non-sonicated fresh CNC suspensions are birefringent, whereas films prepared from suspensions stored several weeks show reflection of left-handed polarized light. Quantification of linear and circular birefringence as well circular dichroism in the films is achieved by using a Mueller matrix formalism.

## 1. Introduction

Cellulose is the most abundant biopolymer on earth. Polymer chains of cellulose conformed by β-1,4 glucopyranose units interact thorough hydrogen bonds leading to formation of semicrystalline fibrils [1]. Nanocellulose materials (nanofibrils and nanocrystals) can be assembled in structured materials and composites (organic or hybrid), with physicochemical properties suitable for biomimicking, sensing, medical, and environmental applications among other fields [2,3,4]. Cellulose nanocrystals (CNC) have been isolated for decades by acid hydrolysis of cellulose fibrils [5,6,7]. With the use of sulfuric acid and by varying CNC concentration, the aqueous suspensions become isotropic, biphasic, or anisotropic; the latter corresponding to a chiral nematic liquid crystal phase [8]. Evaporation-induced self-assembly of CNC suspensions is often used to cast free-standing films [6,9]. In this type of CNC film, the chiral nematic order is retained with a microstructural organization, the so-called Bouligand structure, which is optically expressed as a selective reflection of left-handed circular polarization [10,11]. Among methods to control the spectral position of selective reflection, ultrasound treatment (sonication) of the CNC suspension has proved to produce an increase in pitch and thereby a redshift [12]. A mechanism for sonication-induced increase in pitch was proposed as result of changes in the interactions between CNC and the electrical double layer.

To understand the structural arrangement of CNC in aqueous suspensions, studies of rheological properties have been very valuable. Indeed, effects of concentration, sonication, sulfation degree, and ionic strength on the rheological properties of CNC suspensions have been reported [13,14,15,16]. It has been found that the steady-state viscosity increases with CNC concentration and changes from Newtonian to gel-like (shear-thinning fluid) as the phase changes from isotropic to anisotropic. In contradistinction, the application of ultrasonic power decreases the steady-state viscosity [13]. Recent reviews of the rheology of cellulose nanomaterials account for the importance to process these materials [17,18]. However, to the knowledge of the authors, evidence of changes of viscosity of non-sonicated CNC suspensions over time have not been reported yet. Effects of storage conditions on sulfur content, pH, conductivity, and critical concentration of acidic and neutralized CNC suspensions have been studied before [19,20]. Auto-catalyzed acidic desulfation at 23 °C produced a blue-shift of the wavelength for selective reflection with storage time in free-standing cast CNC films [20]. As is known, this type of film is irregular and other methods should be tested to fabricate flat films for applications in planar configurations.

Since the shear-thinning property of CNC suspensions promotes preferential alignment of nanocrystals, shear-applying methods such as blade coating have been used to produce birefringent films [21,22]. Permanent birefringence in the films was achieved from sonicated CNC suspensions in the anisotropic phase. However, at concentrations of the biphasic regime, the birefringent effect was only temporary because the high mobility of CNC destroyed the alignment [22]. Sheared CNC hydrogels with a large monodomain nematic organization and responsive to pressure and ionic strength suitable as sensors have recently been developed [23]. In addition, methylcellulose/CNC birefringent hydrogels with inverse thermoreversible mechanical stiffening have been reported [24]. The alignment of CNC in dip-coated films from sonicated suspensions of chemically modified CNC has been studied [25]. Additionally, dip-coated mixtures of CNC and carbon-based nanomaterials (nanoparticles, nanotubes, and graphene) for anti-counterfeiting have been reported [26]. Dip coating of non-sonicated CNC suspensions produced birefringent films as reported by our group [27].

Thus, the optical characteristics of nanostructured CNC films, ranging from birefringent to chiral, make them very attractive for applications. As a prerequisite for applications, accurate characterization of the optical response of CNC films must be performed. For this purpose, a Mueller matrix formalism is well suited because it enables methods to evaluate elementary polarization properties of linear and circular birefringence as well as linear and circular dichroism. In nanostructured CNC films, the latter property is related to the structural chirality of CNC in a helicoidal arrangement. In previous studies carried out in our group, the elementary polarization properties of dip-coated [27] and free-standing [28] CNC films were addressed by a Mueller-matrix approach. Additionally, other authors used Mueller-matrix measurements to study the circular dichroism of chiral nematic films of CNC loaded with metallic nanoparticles [29].

In this work, the effect of storage at room conditions of non-sonicated CNC suspensions is studied using polarization optical microscopy (POM) and steady shear viscosity measurements. The use of CNC suspensions without ultrasonic treatment was motivated because an increase in fluidity with storage time was noticed. This indicated a tendency for equilibrium and the change of viscosity in addition to desulfation with time are expected to have an impact on the properties of shear-coated films in a planar configuration fabricated from non-sonicated suspensions. The optical properties of the films are qualitatively evaluated from images recorded by cross-polarizers techniques. The objective is to quantitatively assess the polarization properties of the films, which is performed using Mueller matrices measured by spectroscopic ellipsometry in transmission mode.

## 2. Materials and Methods

### 2.1. Preparation of CNC Suspensions

The preparation of the suspensions followed the procedure previously reported in the literature [7,27]. Ashless filter paper (Whatman 40) was chosen as the source of cellulose. An amount of 8 g was ground using a coffee mill to increase the surface area (3 cycles, 30 s each). The ground filter paper was hydrolyzed with 70 mL of 64 wt% sulfuric acid (J. T. Baker) at 60 °C for 60 min under mechanical stirring. To stop the reaction, 700 mL of distilled water at 5 °C was added and the solution was left to rest for 24 h. The top clear layer was decanted, and the bottom part was centrifuged three times for 10 min each at 9000 rpm to eliminate the hydrolyzed amorphous parts of cellulose. The CNC slurry was dialyzed against distilled water with a dialysis tubing cellulose membrane (Sigma Aldrich) changing the water every 24 h until a neutral pH was reached, which took one week. The concentration of CNC in the dialyzed paste was determined gravimetrically and then diluted in deionized water to a concentration 6.5 wt% and stored at room temperature until used. This concentration was selected because suspensions in the biphasic regime are suitable for fabrication of photonic films with uniform color in larger areas than isotropic or anisotropic phases [30].

### 2.2. Shear-Coating of CNC Films

**Figure 1:**
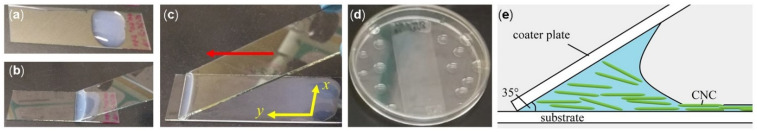
Images of the shear-coating method: (**a**) a drop of CNC suspension is placed on a glass substrate; (**b**) another slide, the coater plate, is placed so that the suspension adheres to it; (**c**) the coater plate is moved to the opposite side of the substrate to distribute the suspension; (**d**) the sample is left inside a Petri dish so that the suspension slowly evaporates in a humid environment. (**e**) Schematics of the deposition of CNC during film deposition.

The films were shear-coated according to the procedure reported by Hoeguer et al. [31], as schematically shown in Figure 1. Glass slides with size 25 × 75 mm^2^ (Corning 2947), washed with water and detergent, were used as substrates and as coater plates. The substrate was placed on a horizontal surface, and a volume of 0.2 mL of CNC suspension was deposited at one end of the glass slide (Figure 1a). The coater plate was then placed at an angle of approximately 35° with respect to the substrate and put in contact with the suspension, which adheres through capillary forces (Figure 1b). Then, the suspension was distributed along the substrate by moving the coater plate as indicated with the red arrow (*y* axis) in Figure 1c at a speed of 5 mm/s. The substrate remained fixed during deposition. The coated substrate was placed inside a Petri dish (90 mm diameter) on a horizontal surface. To promote slow evaporation, water droplets were added around the glass slide and the Petri dish was covered. This procedure for drying has been reported as an effective method to increase the homogeneity of films [30]. The evaporation took place at room temperature for three days.

### 2.3. Characterization Techniques

An Olympus BX60 optical microscope with a Hitachi KP-D50 color digital camera was used to image the samples through crossed polarizers in transmission mode. To observe the fresh suspension, a drop was placed between a glass slide and a cover slip using a 100 μm thick separator. The slides with shear-coated films were placed directly on the microscope stage. Viscosity measurements were performed in a steady-rate sweep test, Couette geometry (cup diameter 16.6 mm, bob diameter 16.2 mm, bob length 14 mm), gap of 10 mm, and shear rates in the range 0.1–100 s^−1^ using an ARES Rheometer (TA Instruments, Inc., New Castle, DE, USA). A dual rotating compensator ellipsometer (RC2, J. A. Woollam Co., Inc., Lincoln, NE, USA) was used to perform Mueller matrix measurements in transmission mode at normal incidence in the wavelength range 210–1690 nm. The diameter of the collimated probe beam was about 3 mm. Film thicknesses were determined from scanning electron microscopy (SEM) images using a JEOL 7610F instrument. To avoid overcharging, a thin layer of Au-Pd was deposited on samples using a Denton Vacuum Desk V with argon as the carrier gas.

## 3. Results and Discussion

Non-sonicated suspensions of CNC prepared under the same hydrolysis conditions and concentration (6.5 wt%) were stored in sealed vials and left at rest under ambient conditions for different periods of time. Evolution of the suspensions properties was observed in a polarization optical microscope and by the viscosity profiles, but also through the optical characteristics of CNC dry films.

### 3.1. Changes with Time in Properties of Non-Sonicated CNC Suspensions

#### 3.1.1. From Birefringent Liquid to Formation of Fingerprint Texture

**Figure 2:**
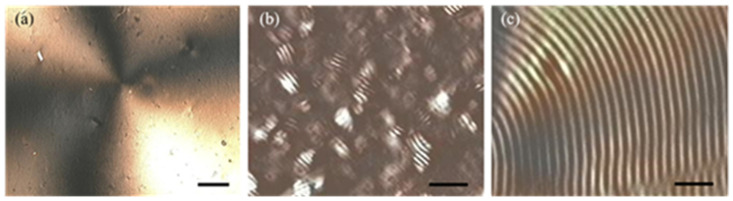
Optical microscopy images of CNC suspensions seen through crossed linear polarizers with a storage time of: (**a**) no storage, (**b**) 90 days, and (**c**) the same sample as in (**b**) but 11 days later. The scale bars are 400, 100, and 20 μm, respectively.

The evolution of non-sonicated CNC suspensions was imaged by POM at different times after preparation, as shown in Figure 2. Figure 2a corresponds to the day the suspension was prepared. Dark and bright areas show linear birefringence generated by the radial orientation that the CNC adopt when the drop is deposited. This texture, characteristic of an anisotropic gel, was observed during approximately two weeks. However, for the CNC concentration used (6.5 wt%) a suspension in the biphasic regime is expected [8], i.e., a coexistence of isotropic and anisotropic phases. The latter phase corresponds to a chiral nematic liquid crystal phase where the director **n**, which defines the preferential orientation of CNC in pseudo-planes, describes a helix. The formation of this type of helicoidal arrangement can be evidenced by the presence of tactoids in POM images, as shown in Figure 2b for a non-sonicated suspension stored for 90 days. In the tactoids, the helical axis is perpendicular to the alternate dark (**n** out-of-plane) and bright (**n** in plane) stripes. The helix pitch determined from the distance between bright stripes for the tactoids observed in Figure 2b was 24.8 ± 1.6 μm. The tactoids in the sample grow with time and finally they form the fingerprint texture characteristic of a chiral nematic liquid crystal phase, as can be seen in Figure 2c. The latter image was taken eleven days after that in Figure 2b. These results indicate that the formation of tactoids in non-sonicated CNC suspensions with a concentration above the critical value requires a certain time to develop. This time can be several weeks as in our case and must be considered during the preparation of chiral films, as will be discussed in Section 3.3.

#### 3.1.2. Changes in Viscosity of Non-Sonicated CNC Suspensions with Storage Time

**Figure 3:**
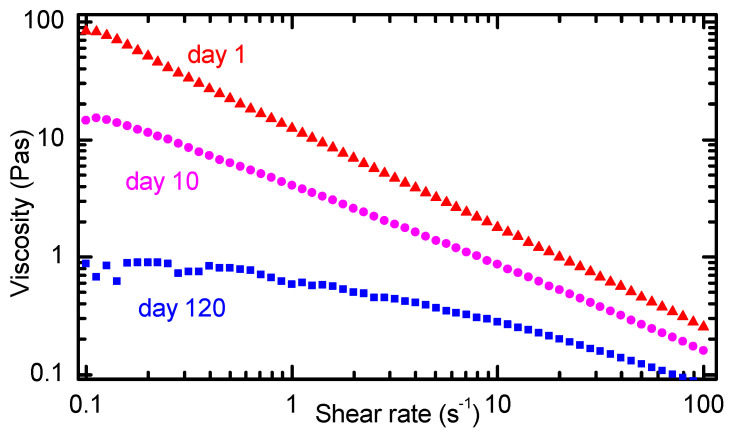
Steady shear viscosity profile of non-sonicated CNC suspensions stored for 1 day (red triangles), 10 days (magenta circles), and 120 days (blue squares).

A key observation during experimentation with non-sonicated CNC suspensions was the increasing fluidity with time. Therefore, the profiles of steady shear viscosity of CNC suspensions were measured at different times after their preparation. Figure 3 shows the dependence of viscosity on shear rate on a logarithmic scale for three measurements made on CNC suspensions with a storage time of 1 day, 10 days, and 120 days. The suspension stored only one day exhibits the typical shear thinning behavior of a gel, which is attributed to the alignment of the CNC in the direction of the applied shear stress [13,16,22]. The gel-like behavior of this non-sonicated CNC suspension agrees with the texture observed in the POM image in Figure 2a. Indeed, other authors have reported that non-sonicated CNC suspensions in the biphasic regime show gel-like viscosity profiles [13]. In Figure 3, it can be noticed that the viscosity profile of the suspension after 10 days of storage also shows the shear-thinning behavior, but with a lower viscosity, which can be noticed at low shear rates. On the other hand, the viscosity profile of the non-sonicated CNC suspension stored for 120 days exhibits a plateau at shear rates below 1 s^−1^ and a shear thinning behavior at higher shear rates. These features in the viscosity profile are characteristic of a chiral nematic liquid crystal phase [17,18].

The steady-shear viscosity profile of a chiral nematic liquid crystalline phase exhibits a three-region profile. Region I at low shear rates is characterized by a shear-thinning behavior attributed to alignment of tactoids in the shear direction. Region II is found at intermediate shear rates where a plateau in viscosity is ascribed to disruption of tactoids into smaller units and release of CNC to some extent. Region III is located at higher shear rates where another shear-thinning zone reflects the breakdown of tactoids and the alignment of the individual CNC in the direction of the applied shear [17,32]. In Figure 3, the viscosity profile of the sample with 120 days of storage shows characteristics of the liquid crystalline phase identified by the plateau below shear rate 1 s^−1^ (region II) and the shear-thinning behavior at higher shear rates (region III). Region I might be located at lower shear rates than the capability of the equipment used. Furthermore, region I has been reported to be elusive and is not always detected [17,33].

According to previous reports [19,20], desulfation of CNC progressively occurs in stored non-sonicated acidic suspensions. The released sulfate groups react with H+ counterions to form sulfuric acid which decreases the pH of the suspensions and increase the conductivity [19,20]. Hence, in non-sonicated suspensions, the screening charge distribution around the twisted-rod CNC might result in a cylindrical effective particle shape and a highly repulsive suspension shows the gel-like behavior [34]. The progressive desulfation over time increases the ionic strength, which decreases the repulsion, and the effective shape changes to a twisted rod, promoting the formation of a chiral nematic liquid crystalline phase [34].

### 3.2. Microstructure of CNC Films Deposited after Different Storage Times

**Figure A1:**
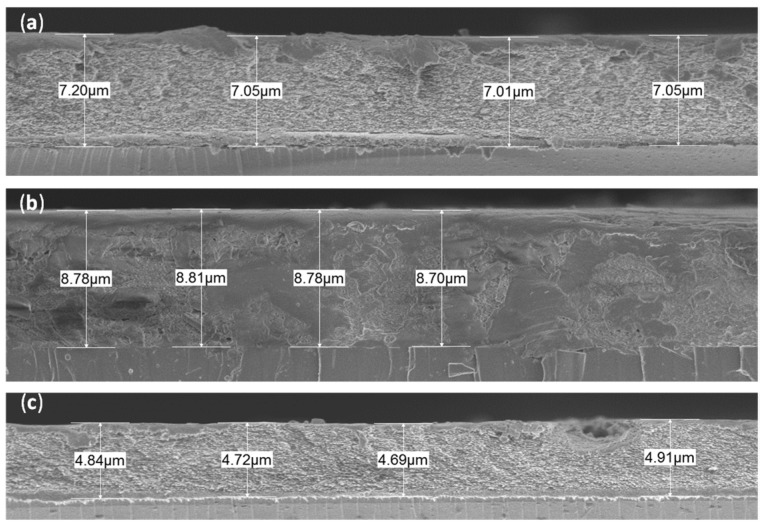
SEM cross-sectional images of films deposited (**a**) on day 1, (**b**) day 18, and (**c**) day 38.

As the non-sonicated CNC suspensions show detectable changes with storage time, shear-coating films were prepared after different times of storage to investigate the effect on microstructure and optical properties of the films. The thicknesses of films prepared at days 1, 18, and 38 of storage determined from SEM images are 7.08 ± 0.08, 8.77 ± 0.05, and 4.79 ± 0.10 μm, respectively (see Figure A1 in Appendix A).

**Figure 4:**
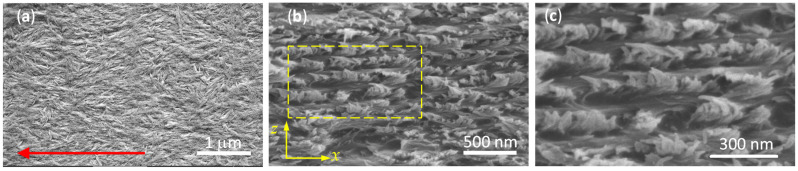
SEM images of shear-coated CNC films: (**a**) the surface of a film prepared on day 1; the arrow indicates the direction of the applied shear. (**b**) Cross section of a film prepared from a suspension stored for 38 days. (**c**) Magnification of the highlighted area in (**b**).

Figure 4a shows a SEM image of the surface of a shear-coated film obtained the same day the non-sonicated suspension was prepared. As was shown in Figure 3, the viscosity of non-sonicated fresh suspensions shows a shear thinning profile (red triangles). The decrease in viscosity with shear rate is ascribed to a preferential alignment of CNC in the direction of the applied shear. In films prepared from this type of suspension, the shear applied during the coating process preferentially aligns CNC, as indicated by the red arrow in Figure 4a.

Figure 4b shows a SEM image of the cross section of a film deposited from a suspension stored for 38 days. The imaged cross section is perpendicular to the shear applied (*x* direction in Figure 1c). The microstructure seen in Figure 4b looks like that often reported for free-standing chiral CNC films [35]. Since CNC films are brittle, a fracture does not always produce a clean cut. The fact that the shear-coated films investigated here are supported on glass (a hard material) introduces another factor that further complicates the possibility to obtain a clean cut. The description of the cross-sectional SEM images is given according to a previous report from other authors [9]. The presence of holes in the structure is explained by the removal of CNC in the opposite section of the fractured film. However, an apparent layered structure of alternate dark and light gray stripes can be noticed, as is shown in Figure 4c, which is a magnification of the highlighted region in Figure 4b. As is known, dried films retain the chiral nematic liquid crystalline phase formed in the suspensions [6]. The dark gray stripes are formed by CNC nearly parallel to the surface of the fracture (*x*-*z* plane), favoring a clean cut. On the other hand, the fracture of the film produces an irregular cut for CNC perpendicular to the cut plane [11]. The latter case is imaged as light gray stripes because the CNC protruding from the fracture surface. The helical axis is parallel to the *z*-axis in Figure 4b.

### 3.3. Optical Characteristics of CNC Films Deposited at Different Storage Times

#### 3.3.1. Birefringent Films from Fresh Suspensions

**Figure 5:**
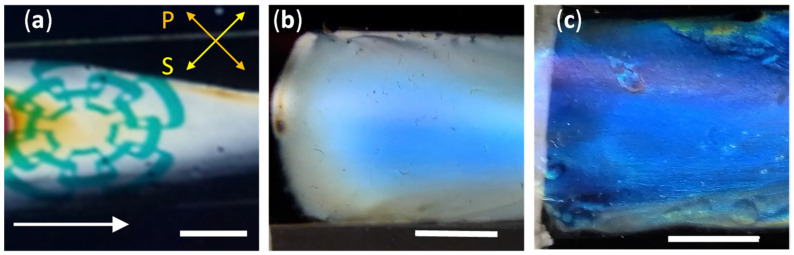
(**a**) Photograph of a film deposited the same day the CNC suspension was prepared, placed at an angle of 45° between a source of linear polarization (S direction) and a crossed polarizer (P direction); the white arrow shows the direction of applied shear. (**b**,**c**) show images of films prepared from non-sonicated suspension stored for 18 and 38 days, respectively. Scale bars are 1 cm.

The film deposited the same day as the CNC suspension was prepared became transparent. However, when viewed through crossed linear polarizers, birefringence was revealed. Figure 5a shows a photograph taken by placing the sample between a liquid crystal display monitor as a source of linearly polarized light at 45° with respect to the horizontal (S direction) and a linear polarizer placed in the extinction configuration (P direction). As can be noticed, the logo of Cinvestav on the screen’s white background is clearly seen. Thicker regions in the film produce the dark yellow color observed in small areas of the sample. In contradistinction to the birefringent shear-coated films of this work, free-standing cast films showed a peak reflection wavelength of 390 nm from non-sonicated suspensions, as reported elsewhere [20].

Other authors have reported preparation of blade-coated CNC birefringent films from sonicated suspensions with CNC concentrations in the anisotropic phase [21,22]. Indeed, in sonicated biphasic CNC suspensions [22], the alignment of CNC was lost after a few minutes of stopping the applied shear because the low viscosity of sonicated suspensions allows the rearrangement of the cholesteric phase. In contradistinction, the larger viscosity of the non-sonicated suspensions in the biphasic regime investigated here locks the alignment of CNC. Microphase transitions in flow towards the nematic order of CNC suspensions showing shear thinning behavior have been reported [36]. However, shear-thinning behavior of CNC suspensions does not ensure alignment of nanocrystals, but a breakup of CNC aggregates might appear [37]. Physicochemical properties of CNC suspensions are largely depending on many factors such as the source of cellulose and hydrolysis conditions, among others [17,18].

#### 3.3.2. Chiral Films from Stored Suspensions

Films deposited from non-sonicated CNC suspensions stored for at least two weeks showed a blue color with only a small linear birefringent effect, as observed when placed between crossed polarizers. Figure 5b shows a photograph of a film obtained from a suspension stored for 18 days as seen through a left-handed circular polarizer. Rotation of the film did not result in any detectable change in the color, which is indicative of a structural origin. Furthermore, selective reflection of left-handed circularly polarized light was confirmed by observation that the film becomes dark when seen through a right-handed circular polarizer. As expected, films deposited for a longer storage time also exhibited selective circular Bragg reflection through tactoid annealing [38]. Figure 5c shows an image of a film obtained from a 38-day-old non-sonicated CNC suspension, also seen through a left-handed circular polarizer. Compared to Figure 5b, a more intense blue color is perceived.

The nucleation of tactoids lead to the coexistence of isotropic and anisotropic phases; i.e., the sample becomes biphasic, as shown in Figure 2b. The biphasic suspension has a viscosity much lower than that of the initial gel. During deposition of the films, the applied shear can lead to deformation of tactoids, high shear rates can align CNC from destroyed tactoids, and some alignment of CNC can be expected. Quantification of chiral and birefringent properties is performed in Section 3.4 below. Recent studies of the relaxation of CNC suspensions after stopping the high shear rate (1000 s^−1^, region III) revealed a buildup of the cholesteric phase through a three-step mechanism in a time scale of minutes [31]: (i) rapid reorganization of CNC in a nematic-like phase, (ii) nucleation and growth of a chiral nematic phase, and (iii) isotropic distribution of the tactoids in the suspension. It is likely that CNC in wet films (Figure 1c) from stored non-sonicated suspensions investigated here follow a similar route to recover the chiral nematic order after deposition. As the drying time is of the order of days, there is enough time for reorganization of CNC to produce the structural color observed in the films.

### 3.4. Quantification of Polarization Properties of CNC Films

The results discussed in previous sections make it very plausible that shear-coated films from non-sonicated CNC suspensions are birefringent or chiral depending on storage time. To quantify the polarization properties of a sample, a Mueller matrix formalism is well suited, and is employed in this section. Normal incidence transmission measurements of Mueller matrices were performed; thus, propagation of light is along the *z*-axis, as defined in Figure 4b.

#### 3.4.1. Mueller Matrices of CNC Films Measured in Transmission

A complete description of the polarization and depolarization properties of a linear optical system is given by the Stokes–Mueller approach [39]. In this approach, light beams are represented by column vectors **S** = [*I*,*Q*,*U*,*V*]^T^ (T means transpose) where *I* represents the total irradiance; *Q* > 0 (<0) accounts for the tendency for linear polarization along the *x*-axis (*y*-axis)*; U* > 0 (< 0) for the tendency for linear polarization along direction +45° (−45°) in the *xy*-plane; and *V* > 0 (<0) for the right (left) circular character of polarization. After interacting with a system, the Stokes vector of the resulting beam is given by **S**’ = **MS** where **M** = {*M_ij_*} is the 4 × 4 Mueller matrix of the system. In this work, normalized Mueller matrices (*m_ij_* =*M_ij_*/*M*_11_) and Stokes vectors (*I* = 1) are used.

**Figure 6:**
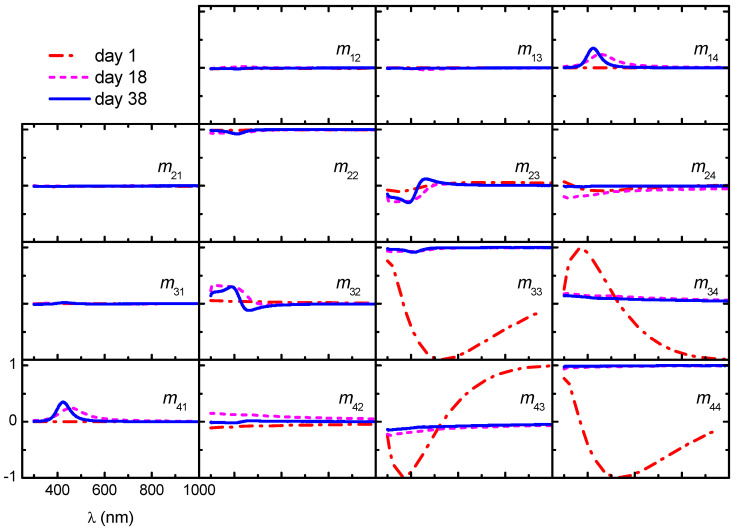
Evolution of Mueller matrices in transmission mode of films deposited from CNC suspensions with different times of storage. Scales are shown in the lower-left panel.

Figure 6 shows Mueller matrices measured on samples fabricated from non-sonicated CNC suspensions stored at different times. Data for the film fabricated with the fresh (day 1) suspension have most of the elements close to zero with the exemption of *m*_22_ (≈1) and the 2 × 2 lower-right block. This is indicative of a sample with linear birefringence with the slow and fast axes aligned with the laboratory frame (*x* − *y*) [39].

A drastic change in the Mueller matrices of films prepared from stored non-sonicated suspensions is noticed: the 2 × 2 lower-right block becomes nearly a unity block, whereas the elements in the secondary diagonal show structure below wavelength 600 nm; other elements are close to zero. In data of the film prepared from the 18-day-old suspension, *m*_41_ exhibits a band centered at wavelength 455 nm, whereas in the film prepared with a 38-day-old suspension, the band is at 423 nm. The presence of these bands is consistent with the blue color of the films and the blue-shift with storage time agrees with the result in cast films [20]. For unpolarized light, which is described with a Stokes vector **S** = [1,0,0,0]^T^, the transmitted beam is given by **S’** = [1,*m*_21_, *m*_31_, *m*_41_]^T^. Since *m*_21_ and *m*_31_ are nearly zero and *m*_41_ > 0, films obtained from stored suspensions transmit right-handed polarized light; thus, left-handed polarized light is reflected.

**Figure 7:**
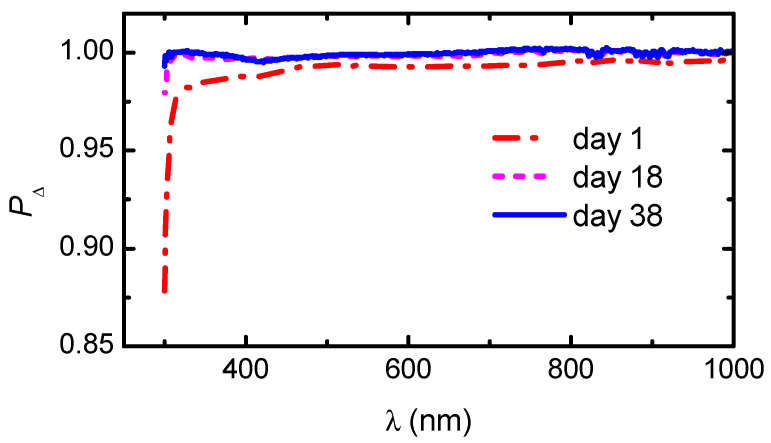
Depolarization index of the Mueller matrices shown in Figure 6.

From a Mueller matrix, the linear optical response to any incident beam can be determined, regardless of its polarization state. This means that the degree of polarization, ellipticity, and azimuth of the polarization ellipse of the emerging beam can be quantified [39]. However, of high interest are properties of the sample related to its micro- and nanostructure and intrinsic properties of the constituent materials. In the present case, there is preferential alignment of CNC in birefringent films and a helicoidal arrangement of CNC in structurally colored films. To determine sample properties, the capability of the system to depolarize incident polarized light should be evaluated first. A measure of this capability is given by the polarization index *P*_Δ_ of a normalized Mueller matrix **M** [40],

(1) $$P_{\Delta }=\sqrt{\frac{\mathrm{tr}M^{T}M-1}{3}},$$

where tr stands for trace. A value of *P*_Δ_ = 0 corresponds to an ideal depolarizer and *P*_Δ_ = 1 to a non-depolarizing system. Figure 7 shows *P*_Δ_ for films prepared from non-sonicated suspensions stored for 1, 18, and 38 days, where the average values of *P*_Δ_ are 0.993 ± 0.008, 0.999 ± 0.002, and 0.999 ± 0.001, respectively. In conclusion, for measurements in transmission mode, the CNC films practically do not depolarize incident polarized light.

#### 3.4.2. Polarization Properties of CNC Films

With polarization properties, we refer to linear birefringence (linear diattenuation) referred to the *x* − *y LB* (*LD*) and ±45° *LB’* (*LD’*) frames as well as circular dichroism *CD* and circular birefringence *CB*. *LB* and *LB’* are related to the difference in refractive indices for electric field parallel and perpendicular to the direction of the applied shear during deposition. As the optical principal axes of the sample in general are not aligned with the laboratory frame, but deviate by an angle *φ*, it is convenient to use *LB* = *δ*cos*φ* and *LB’* = *δ*sin*φ,* where *δ* is the retardation introduced by the sample. Differences in absorption in *x*-*y* and ±45° directions account for *LD* and *LD*’, respectively. Similarly, *LD* = *p*cos*φ* and *LD’* = *p*sin*φ* where *p* is the diattenuation. The difference in speed of propagation of left- and right-handed circular polarization states in the sample is contained in *CB*. *CD* is related to sample selectivity for transmission of left- and right-handed circular polarization. There exist several methods for an accurate determination of these polarization properties of a sample from its experimental Mueller matrix. Since the films investigated in this work correspond to non-depolarizing samples (*P*_Δ_ ≈ 1 in Figure 7), analytical inversion is suitable [41], whereby *LB*, *LD*, *LB’*, *LD’*, *CD*, and *CB* are obtained explicitly in terms of the Mueller matrix elements *m_ij_*. To eliminate the orientational dependence (*φ*), the retardation *δ* = (*LB*^2^ + *LB*’^2^)^1/2^ and diattenuation *p* = (*LD*^2^ + *LD*’^2^)^1/2^ are then calculated.

**Figure 8:**
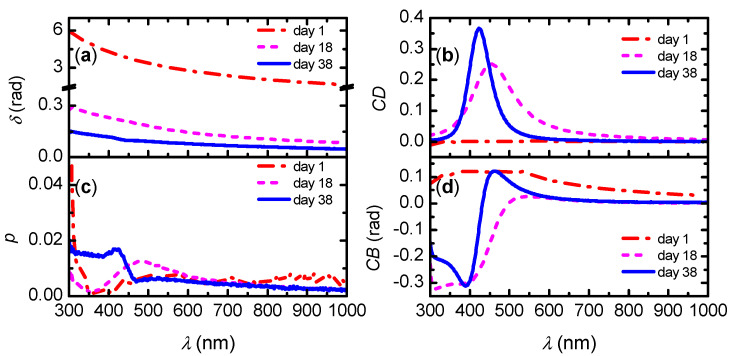
Polarization properties of CNC films prepared from non-sonicated suspensions stored for the indicated number of days: (**a**) retardation *δ*, (**b**) circular dichroism *CD*, (**c**) attenuation *p*, and (**d**) circular birefringence *CB*. Notice the change of scale in the vertical axis in (**a**).

Figure 8 shows the polarization properties of the films prepared from non-sonicated suspensions stored for different periods of time. As can be seen in Figure 8a, the film prepared from fresh non-sonicated suspensions has high retardation. This property is largely diminished in films obtained from stored suspensions. On the other hand, *CD* shows an opposite behavior with storage time, as seen in Figure 8b. *CD* is nearly zero at day 1 and a band is developed in films prepared from stored suspensions. In all cases, the linear diattenuation is very low (Figure 8c), which means poor performance as a linear polarizer as expected. The Kramers–Kronig consistency of *CD* and *CB* noticed in Figure 8b,d is also expected. To the authors’ knowledge, *CB* of CNC chiral films is not often reported, with the exemptions of our previous works on free-standing [30] and shear-coated CNC films [42]. In the latter work, a quantification of the chirality parameter using Tellegen constitutive equations confirmed the Kramers–Kronig consistency.

## 4. Conclusions

Non-sonicated CNC suspensions with concentration in the biphasic regime show a gel-like state. Storing of the suspensions at room temperature conditions largely decreases their viscosity with time and evolves from a viscous birefringent gel to a sample with a chiral nematic liquid crystal phase. This evolution of the viscosity with storage time might be explained by the effects of the progressive desulfation of CNC [19,20]; change in the screening charge distribution around the twisted-rod CNC from a highly repulsive to a chiral nematic suspension was promoted by the increasing ionic strength. The structure of the non-sonicated CNC suspensions is retained in dried shear-coated films. Birefringent films are obtained from fresh suspensions and chiral films from stored suspensions. Linear and circular birefringence as well as circular dichroism of the CNC films were accurately quantified from Mueller matrix data.

## Data Availability

The data presented in the study are available in the article (figures).
